# Social exclusion alters attention and autonomic regulation in adolescents with nonsuicidal self-injury

**DOI:** 10.1038/s41398-026-04136-w

**Published:** 2026-05-24

**Authors:** Andreas Goreis, Annika Lozar, Rosa List, Sofia-Marie Oehlke, Bettina Pfeffer, Karin Prillinger, Diana Klinger, Heidi Zesch, Peter B. Marschik, Laurence Claes, Paul L. Plener, Oswald D. Kothgassner

**Affiliations:** 1 Department of Child and Adolescent Psychiatry, Medical University of Vienna, Vienna, Austria; 2 Comprehensive Center for Pediatrics (CCP), Medical University of Vienna, Vienna, Austria; 3 Comprehensive Center for Clinical Neuroscience and Mental Health (C3NMH), Medical University of Vienna, Vienna, Austria; 4 Department of Child and Adolescent Psychiatry, University Hospital Heidelberg, Heidelberg University, German Center for Mental Health (DZPG), Heidelberg, Germany; 5 Interdisciplinary Developmental Neuroscience (iDN), Department of Neurology, Medical University of Graz, Graz, Austria; 6 Center of Neurodevelopmental Disorders (KIND), Department of Women’s and Children’s Health, Centre for Psychiatry Research, Karolinska Institutet & Region Stockholm, Stockholm, Sweden; 7 Child and Adolescent Psychiatry and Psychotherapy, University Medical Center, German Center for Child and Adolescent Health (DZKJ), Göttingen, Germany; 8 Clinical Psychology, Faculty of Psychology and Educational Sciences, KU Leuven Leuven, Belgium; 9 Faculty of Medicine and Health Sciences, University of Antwerp, Antwerp, Belgium; 10 Department of Child and Adolescent Psychiatry and Psychotherapy, University of Ulm, Ulm, Germany

**Keywords:** Human behaviour, Physiology

## Abstract

Nonsuicidal self-injury (NSSI) poses a growing clinical challenge in adolescence, while imagery of NSSI is increasingly embedded in everyday digital life. Prior research indicates that adolescents who engage in nonsuicidal self-injury orient more rapidly toward NSSI-related imagery and report heightened urges to self-injure after viewing such content. What remains unknown is how social stress shapes these attentional biases. In the present study, we tested whether experimentally induced ostracism modifies attention to NSSI images, urges to self-injure, and autonomic regulation in adolescents with recent NSSI. Fifty adolescents who engaged in recent NSSI (*M* = 16.40 years, *SD* = 1.33) were randomized to inclusion vs. exclusion in an in-person ball-tossing ostracism paradigm and then completed free-viewing eye-tracking (with stimuli durations of 500/1000 ms) and dot-probe tasks (200/500 ms) that included NSSI images. NSSI urges, perceived stress, heart rate, and heart rate variability (Root Mean Square of Successive Differences, RMSSD) were assessed throughout the experiment. Social exclusion produced sustained reductions in RMSSD relative to inclusion, with no reliable heart rate differences. During free-viewing, both groups preferentially fixated NSSI images, but exclusion reduced the probability of first fixations on NSSI. In the dot-probe task, exclusion amplified the slowing from congruent to incongruent trials for NSSI. After viewing NSSI images, both groups showed increased urges and stress, with larger increases after exclusion. Acute interpersonal rejection reorganized attention, dampening initial gaze capture yet impairing later disengagement, occurring in the context of vagal withdrawal. These dynamics may help explain how social stress potentiates NSSI risk in everyday digital environments and highlight interpersonal context and post-rejection coping as modifiable targets for intervention. **Trial Registration**: German Clinical Trials Register Identifier: DRKS00025905

## Introduction

Nonsuicidal Self-Injury (NSSI) is the deliberate, repeated infliction of bodily harm without suicidal intent, typically cutting, scratching, burning, or hitting oneself [1]. As a transdiagnostic entity that frequently co-occurs with depressive disorder, posttraumatic stress disorder (PTSD), and borderline personality disorder (BPD) [2–4], NSSI is listed in Section III of the DSM-5 as a condition warranting further study, defined as self-harm on at least five days within the past year [5]. A systematic review of longitudinal studies [6] found that NSSI prevalence peaks at ages 15–16 and declines by age 18. Nevertheless, the behavior remains common, with global prevalence estimates of 16–22% among adolescents, and higher rates reported in females than males [7, 8]. Rising rates of NSSI have been reported in studies in recent years [9]. Critically, NSSI is also a significant predictor of later suicide attempts [10].

Given the ubiquity of social media in adolescence [11], NSSI/self-harm imagery is easily encountered and shared [12]. Emerging work indicates that such exposure can trigger NSSI urges and behaviors [13]. A recent review reports predominantly harmful effects of viewing self-harm images online [14]. Beyond intrapersonal antecedents (e.g., exposure to images), research identifies interpersonal stressors (e.g., criticism, conflict, and rejection) as proximal risks for NSSI [15, 16], including when they occur in digital settings [17, 18]. Foundational models of NSSI [19, 20] posit that interpersonal stressors (e.g., social rejection) interact with intrapersonal vulnerabilities (e.g., emotion dysregulation, heightened stress reactivity) to increase NSSI urges and precipitate self-injury, particularly among those with prior NSSI.

In a laboratory eye-tracking study focusing on the precipitating effects of online NSSI imagery [21], we found that adolescents with recent NSSI—but not healthy controls—reported increased urges and perceived stress after viewing NSSI-related images resembling those commonly encountered on social media. They also showed an attentional bias toward NSSI cues, indexed by faster initial orienting and longer fixation durations. Although laboratory paradigms differ from everyday contexts, these effects have practical implications: adolescents routinely encounter NSSI content across feeds, private messages, and search results [22], contexts in which attention may be captured and urges rise. Convergent evidence from dot-probe tasks—a reaction-time paradigm for assessing attentional bias—likewise supports the presence of this bias in adolescents [21] and adults [23]. In these tasks, two images are presented briefly, followed by a probe (e.g., a white dot); attentional bias is indexed by slower responses on incongruent trials (when the probe appears opposite the NSSI image) because attention must disengage from the NSSI location and reorient to the opposite side. Taken together, adolescents with an NSSI history show selective attentional capture by NSSI imagery that co-occurs with heightened urges.

Adolescence is also marked by heightened sensitivity to social rejection [24]. In adolescents who engage in NSSI, rejection episodes are strongly aversive, degrade momentary well-being, and are associated with increased NSSI urges and acts as well as suicidal thoughts and behaviors [17, 25, 26]. It has been shown that social exclusion leads to enhanced neural activation in the ventral anterior cingulate cortex in adolescents with NSSI [22], and that these adolescents also show a blunted cortisol response during social stress tasks [27]. Converging evidence also implicates autonomic stress regulation during social rejection [28], typically characterized by increased sympathetic activation and parasympathetic withdrawal [29].

What remains unclear is whether rejection not only elevates affective risk but also modulates cognitive mechanisms implicated in NSSI—specifically, the temporal dynamics of attentional bias to NSSI-related imagery. In the vigilance–avoidance theory of cognitive processing [30], early orienting reflects rapid gaze capture (e.g., initial fixations), whereas later-stage attentional processing reflects sustained engagement and disengagement difficulty once attention is allocated. Prior experimental evidence in adolescents with NSSI [21] is consistent with this distinction, showing rapid orienting to NSSI stimuli alongside evidence of prolonged engagement. Importantly, effects of social exclusion on attentional bias may be stage-dependent rather than uniformly directional. Thus, exclusion may influence early orienting and later-stage disengagement differently, rather than simply heightening attention toward NSSI cues. Building on this work, we tested whether social rejection alters early orienting (gaze capture) and/or later-stage attentional processing (including disengagement-related indices) and how these changes unfold alongside autonomic regulation and momentary urges to self-injure.

In the present study, we experimentally manipulated social inclusion vs. exclusion using an ostracism paradigm (i.e., Cyberball [31]) adapted to live interaction with real humans: a three-player ball-toss task in which two confederates either included or excluded the participant according to a scripted sequence. This adaptation was made because traditional computerized Cyberball paradigms have been criticized for their lack of realism and ecological validity [32]. Following the ostracism task, adolescents with recent NSSI completed (1) an eye-tracking free-viewing task to index early orienting (first-fixation probability, time to first fixation) and sustained engagement (fixation duration) for NSSI vs. neutral images, and (2) a dot-probe task to index disengagement-related attentional efficiency (incongruency cost). Across the experiment, we assessed subjective stress and NSSI urges, as well as autonomic activity via heart rate and heart rate variability indexed by the root-mean-square of successive differences (RMSSD).

We hypothesized that social exclusion would (1) increase subjective stress and NSSI urges and shift autonomic activity—specifically, increase heart rate and decrease heart rate variability (RMSSD); (2) heighten attentional capture by NSSI images during free-viewing, reflected in a higher probability of first fixations on NSSI images and shorter times to first fixation (early orienting), and increase sustained engagement, reflected in longer fixation durations; (3) heighten dot-probe attentional bias for NSSI stimuli—i.e., larger incongruency effects (slower responses when the probe appears opposite the NSSI image compared with congruent trials), consistent with greater disengagement-related costs; and (4) increase subjective stress and NSSI urges in response to exposure to NSSI images, with stronger increases following exclusion.

## Methods

This study was registered in the German Clinical Trials Register (identifier: DRKS00025905), performed in accordance with the Declaration of Helsinki, and approved by the ethics committee of the Medical University of Vienna, Austria (protocol number: 1651/2019). Written informed consent was obtained from participants and their legal guardians before participation.

### Participants

Fifty adolescents (aged 14–18 years) with a history of ≥5 NSSI episodes in the preceding 12 months (according to the DSM-5 frequency criterion) were enrolled between 18 December 2023 and 24 March 2025. Additional inclusion criteria were absence of autism spectrum disorder (to avoid eye-tracking calibration/interpretation issues that could compromise data quality); absence of severe medical or psychiatric conditions necessitating acute treatment (e.g., psychosis), pronounced aggression, or acute suicidality. All participants were fluent in German. Participants were recruited primarily via the in- and outpatient services of the Department of Child and Adolescent Psychiatry at the Medical University of Vienna, Austria, with supplementary enrollment through social-media advertisements and referrals from external psychological and psychiatric practitioners.

A total of 107 adolescents with NSSI were screened for inclusion. Of these, 27 participants were excluded due to nonresponse or loss of interest, and 12 because their legal guardians did not provide written consent. Consequently, 68 participants with NSSI were invited to participate in the study; 18 did not attend their scheduled appointment resulting in a final sample of 50 participants. An a priori power analysis (G*Power [33]) indicated that a total sample of *N* = 50 (25 per condition) provides 80% power (α = 0.05, two-sided) to detect medium-sized effects in our primary experimental contrasts, most centrally condition-related differences over time (i.e., Condition × Time effects in repeated-measures outcomes) and condition differences in attentional indices. We specified Cohen’s f = 0.25 as a pragmatic benchmark for effects considered clinically and theoretically meaningful in stress responding and attentional processing in laboratory paradigms. Following a minimal-principle approach, and accounting for the typical nonattendance and attrition in clinical adolescent samples, recruitment continued until 50 participants completed the laboratory protocol, as pre-specified in the trial registration.

### Procedure

**Fig. 1:**
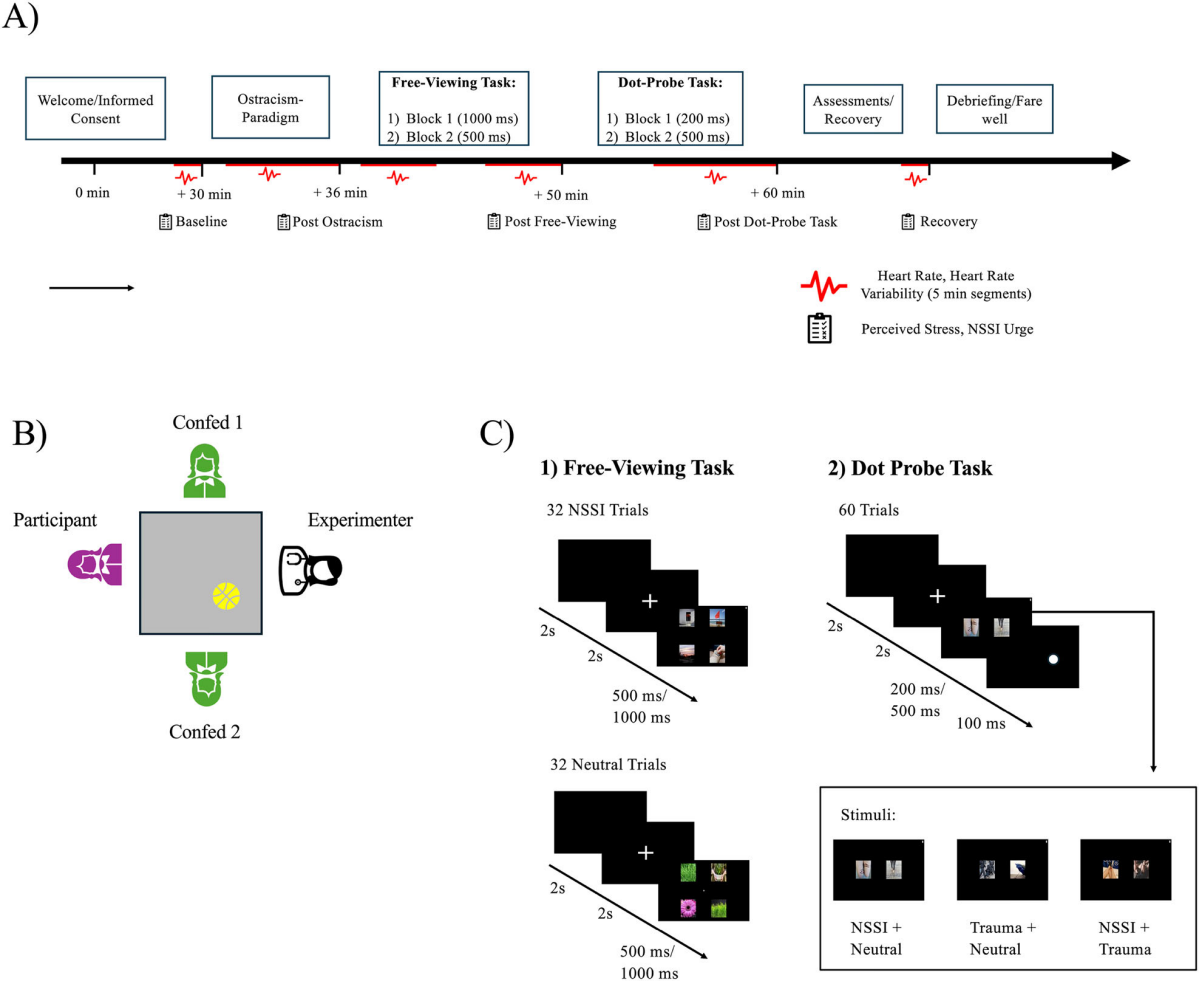
Study design and experimental tasks. **A** Overview of study procedure and timeline, **B** Schema of the in-person ball-tossing ostracism paradigm, **C** Overview of the free-viewing and the dot-probe task.

The study was conducted at the Department of Child and Adolescent Psychiatry at the Medical University of Vienna, Austria. Initially, heart-rate monitors were affixed, and participants underwent a 30-min baseline phase in a quiet laboratory setting to acclimate and complete the study questionnaires. Following the baseline, they were escorted to an adjacent room to complete the 6-min ostracism paradigm. Subsequently, participants were guided back to the first room, seated at 60 cm from a Tobii TX-Display eye-tracking monitor, and given task instructions. The attentional-bias assessment commenced immediately with the free-viewing task, followed by the dot-probe task. Momentary NSSI urges and stress were assessed using 5-point Likert scales (ranging from 1, not at all, to 5, extremely) at five time points (Fig. 1A). Participants were then debriefed, thanked, and given a €25 voucher.

### Ostracism paradigm

Participants took part in an in-person ball-tossing ostracism paradigm (conceptually based on the Cyberball paradigm [31]) in which they sat in a chair at one end of an 80 ×80 cm wooden table opposite the experimenter, with two female confederates (research assistants/psychology students whom they believed to be fellow participants) seated on either side (Fig. 1B). Using a standard tennis ball, all three players were verbally instructed by the experimenter (following a standardized script) to roll the ball across the table to one another as quickly and accurately as possible while the experimenter monitored time with a stopwatch. After an initial one-minute fair-play phase, a covert beep (from the stopwatch) signaled the start of the randomized five-minute condition: in the inclusion condition, the confederates continued to toss the ball equitably; in the exclusion condition, they delivered three more throws to the participant before deliberately excluding them for the remainder of the session.

Condition assignment was randomized in advance by a research assistant; participants remained blind to it, while only the two confederates were informed whether to include or exclude the participant after the covert signal (which was always given by the experimenter after the first minute). Prior to data collection, the confederates completed training with rehearsal of the inclusion and exclusion scripts and instructions to minimize non-task social cues, including no conversation, minimal eye contact, and neutral facial expression and gestures. The experimenter was blind to the assigned condition until the signal, after which he could observe how the game unfolded. At the end of the six-minute paradigm, the experimenter escorted the participant to an adjacent room to complete self-report validity checks, percentage of tosses received, perceived ignoring/exclusion during the game, and the Reflexive Needs Questionnaire [31], after which the eye-tracking free-viewing task began.

### Free-viewing eye-tracking task

The free-viewing task comprised two blocks of 64 trials (Fig. 1C). In Block 1, each trial presented four images for 500 ms: 32 trials contained one NSSI-related and three neutral images, and 32 trials contained four neutral images. Block 2 used the same trial structure with a 1000 ms presentation. All trials began with a 2-second black screen and a 2-second fixation cross (Fig. 1C). Stimuli were randomized within blocks, with NSSI-related images evenly distributed across positions. Seventy-five percent of NSSI images were self-generated (SFX make-up) by one of the authors (BP; White, cisgender female), depicting shards of glass, razors, and artificial blood both alone and on limbs, and the remainder were drawn from publicly available social-media posts to enhance ecological validity. The NSSI stimulus set was selected to resemble NSSI-related imagery commonly encountered in adolescent social-media contexts [12, 17]. To reduce low-level visual confounds, all stimuli were standardized in presentation format (size/resolution), balanced across screen positions, and 25% of both NSSI-related and neutral images were presented in grayscale to reduce color-driven salience differences that may influence attentional capture independent of stimulus content. Neutral images were sourced from the International Affective Picture System (IAPS [34]). Given the limited availability of validated, normed NSSI image sets, we assembled the NSSI stimulus set to maximize ecological validity while applying these standardized presentation controls. We used the same stimulus set and task parameters as in Goreis et al. [21], where this paradigm elicited robust attentional-bias indices and urge reactivity in adolescents with NSSI, supporting its construct validity. All stimuli are publicly accessible (10.17605/OSF.IO/6PJRN).

### Dot-probe task

The dot-probe task, based also on Goreis and colleagues [21], comprised 60 image pairs: 20 NSSI–neutral, 20 trauma–neutral, and 20 NSSI–trauma (Fig. 1C). Each pair appeared twice (200 ms in Block 1 and 500 ms in Block 2) using the same NSSI and neutral images from the free-viewing task, and trauma images (e.g., gun, strangulation, medical emergency) were taken from the IAPS [34] and included as a non-self-injury-related, threat-related high-arousal comparison category, allowing us to test whether potential effects were specific to NSSI cues rather than reflecting a general threat-related/aversive attentional bias. Twenty-five percent of image pairs were shown in grayscale. Each trial began with a 2-s fixation cross, followed by the image pair flanking the former cross position. After 200 ms (Block 1) or 500 ms (Block 2), images vanished and a dot (the probe) appeared for 100 ms in the location of one image. Participants pressed a left or right arrow key (handedness-adjusted keyboard) as quickly and accurately as possible. Stimulus side was counterbalanced (50% left/right) within all conditions.

Trials in which the probe replaced an NSSI or trauma image in the first two conditions were “congruent” (engagement), vs. “incongruent” when it replaced the neutral image (disengagement). In NSSI + trauma trials, a probe at the NSSI location was congruent and at the trauma location incongruent. Faster responses to probes at prior NSSI vs. neutral (or trauma) locations indexed vigilance; slower responses to probes at neutral or trauma vs. NSSI locations indexed disengagement difficulty. Before the main task, participants completed 10 neutral practice trials (five at 200 ms, five at 500 ms).

### Measures

#### Nonsuicidal Self-Injury (NSSI)

The short form of the German version of the Self-Injurious Thoughts and Behavior Interview (SITBI-R) [35] was used for the assessment of the participants’ history, frequency and prevalence of NSSI.

#### Depressive symptoms

We assessed depressive symptoms by employing the German version of the Beck Depression Inventory II (BDI-II) [36]. This self-report questionnaire consists of 21 individual items which are to be rated on a scale ranging from 0 to 3. Higher scores indicate more depressive symptomatology. Reliability of the BDI-II was Cronbach’s α = 0.91 in this sample.

#### Perceived stress

Perceived stress was assessed with the German version of the Perceived Stress Scale (PSS-10) [37]. The 10 items ask how unpredictable, uncontrollable, and overwhelming daily life felt, using a 5-point scale (0 = never, 4 = very often); higher total sum scores denote greater stress. Reliability was α = 0.80.

#### Post-Traumatic Stress Disorder (PTSD) symptoms

Due to the high comorbidity of NSSI and PTSD, we screened all participants for PTSD symptoms. Thus, the German version of the Child and Adolescent Trauma Screen (CATS-2) [38], a self-report measure for children and adolescents, was used. The CATS-2 comprises 25 items assessing severity of PTSD symptoms based on both ICD-11 and DSM-5 criteria. All items are rated on a 4-point Likert scale. Evaluation for PTSD was based on DSM-5 criteria, i.e., scores ≥25 were used as screening thresholds for the presence of PTSD. Reliability was α = 0.88.

#### Borderline Personality Disorder (BPD) symptoms

Given the high comorbidity of NSSI with BPD, the Borderline Symptom List-23 (BSL-23) [39] was administered to screen participants for BPD symptoms. This self-report measure entails 23 items which are to be rated on a 5-point Likert scale (ranging from “not at all” to “very strongly”) and assesses severity of BPD-related symptoms. Reliability was α = 0.89.

#### Reflexive needs questionnaire

The Reflexive Needs Questionnaire [31] assessed participants’ feelings of belonging, self-esteem, control, meaningful existence (five items each), and mood (eight items) during the ostracism task. Participants rated the extent to which each statement reflected their feelings during the game (e.g., “I felt rejected,” “I felt good about myself,” “I felt powerful”; mood items included “I felt good/happy/sad”) on a 5-point Likert scale from 1 (not at all) to 5 (extremely). The questionnaire was administered immediately after the ostracism task; internal consistency of the total score was α = 0.93.

#### Initial fixation and fixation durations

In the free-viewing task, fixations were defined as continuous gazes within a stimulus (neutral or NSSI image) lasting ≥100 ms. Initial fixation was operationalized as the first stimulus to which gaze was directed after trial onset. We derived three indices of early attention: (1) first fixation probability, the proportion of trials with initial fixations on NSSI images; (2) time to first fixation, the latency from trial onset to the first fixation; and (3) first fixation duration, the time spent on the initial fixation before shifting away.

#### Electrophysiological measures

Heart rate was used as a marker of sympathetic activity, and RMSSD indexed short-term parasympathetic modulation of heart rate variability. Cardiac signals were continuously recorded with a movisens EcgMove 4® sensor (movisens GmbH, Karlsruhe, Germany; 1024 Hz). The two-electrode sensor was placed below the sternum, and ECG recordings were converted into beat-to-beat heart rate (bpm) and RMSSD (ms) using DataAnalyzer® software (movisens GmbH). Five-minute segments were analyzed at six time points: baseline (30 min after arrival), during inclusion or exclusion in the ostracism paradigm, during the free-viewing task (separately for 500 and 1000 ms blocks), during the dot-probe task, and 20 min post-dot-probe (recovery). To minimize posture- and movement-related confounds, participants remained seated throughout all ECG recording segments (ostracism, computerized tasks, and recovery).

### Statistical analyses

All analyses were conducted in R (version 4.4.2) using the packages lme4 for mixed-effects modeling, lmerTest for *p*-values and degrees of freedom (Satterthwaite approximation), emmeans for estimated marginal means and Tukey-adjusted contrasts, and ggplot2 for visualization. We used independent-samples *t*-tests for ostracism manipulation checks. For longitudinal physiological outcomes (heart rate, RMSSD) and self-report outcomes (NSSI urge, stress), we fit linear mixed-effects models with condition (exclusion vs. inclusion) as a between-subjects factor and time as a within-subjects factor. In the free-viewing task, models included condition (between) and stimulus type (NSSI-related vs. neutral). Initial fixation probability was analyzed with a binomial generalized linear mixed-effects model (logit link); time to first fixation and fixation duration were analyzed with linear mixed-effects models. For the dot-probe task, mixed-effects models included condition (exclusion vs. inclusion) as between-subjects factor, and stimulus type (NSSI vs. trauma), and probe congruency (congruent vs. incongruent) as within-subjects factors. Across all mixed-effects analyses, observations at Level 1 were nested within participants at Level 2, and we specified participant ID as random intercepts in all models (model diagnostics are reported in the Supplementary Material). Missing data were not imputed because mixed-effects models can be applied in case of missing or unequal data. Two-sided tests with α = 0.05 defined statistical significance.

Post-hoc pairwise contrasts were Tukey-adjusted within each model to control multiplicity for planned comparisons. Given that we analyzed multiple outcomes across tasks, we did not apply an additional global correction across all models. Instead, we interpreted results within pre-specified outcome families (self-report, physiology, eye-tracking, dot-probe) and prioritized effect sizes and confidence intervals, because an across-model correction would be overly conservative for our hypothesis-driven tests and would inflate Type II error. For effect-size standardization, we reported Hedges’ *g* for between-group comparisons without repeated measures (*g* = 0.20/0.50/0.80 indicating small/medium/large effects) and Nakagawa’s marginal *R*^2^_m_ for mixed-effects models (proportion of variance explained by fixed effects).

## Results

The final sample comprised 50 participants (*M* = 16.40 years, *SD* = 1.33); 38 identified as female, and all identified as Caucasian (see Table 1 below). Twenty-five were randomized to the ostracism paradigm inclusion condition and 25 to the exclusion condition. On average, participants reported 1.67 NSSI episodes in the past week, 6.40 in the past month, and 114.52 in the past year. The most common methods were cutting (97.9%), scratching (81.3%), punching (70.8%), burning (58.3%), biting (52.1%), and inserting objects under nails or skin (17%). CATS-2 scores based on the DSM-5 algorithm indicated, on average, probable PTSD (*M* = 34.73, *SD* = 11.65). Depressive symptoms (*M* = 32.78, *SD* = 12.94), borderline symptoms (*M* = 50.73, *SD* = 23.19), and perceived stress (*M* = 25.75, *SD* = 7.34) were also elevated. At the time of testing, 46% of participants were on psychotropic medication. No group differences were observed in baseline characteristics (all *t*- and *χ²*-tests: *p* > 0.212), confirming successful randomization. Table 1 provides an overview of sample characteristics and ICD-10 diagnoses (total and by condition).

**Table 1:** Participant Characteristics.

Characteristic	Mean (*SD*)		
	Total Sample	Inclusion Condition	Exclusion Condition
Age (years)	16.40 (1.33)	16.32 (1.41)	16.48 (1.26)
Age (years) first time NSSI	12.08 (2.25)	12.04 (1.57)	12.13 (2.80)
NSSI 1-week prevalence (SITBI-R)	1.67 (3.75)	2.00 (5.03)	1.33 (1.79)
NSSI 4-week prevalence (SITBI-R)	6.40 (7.49)	6.13 (6.96)	6.67 (8.12)
NSSI 1-year prevalence (SITBI-R)	114.52 (173.57)	85.38 (114.18)	143.67 (216.22)
PTSD-symptoms (CATS-2, DSM-5)	34.73 (11.65)	33.13 (11.04)	36.33 (12.25)
Perceived Stress Scale-10	25.75 (7.34)	24.80 (6.71)	26.44 (7.78)
Beck Depression Inventory II	32.78 (12.94)	30.48 (12.35)	35.08 (13.35)
Borderline Symptom List-23	50.73 (23.19)	45.76 (21.06)	53.56 (25.92)
	***N*** **(%)**	***n*** **(%)**	***n*** **(%)**
Gender			
Female	38 (76%)	21 (84%)	17 (68%)
Male	3 (6%)	1 (4%)	2 (8%)
Gender-diverse	9 (18%)	3 (12%)	6 (24%)
Current psychotropic medication	23 (46%)	12 (48%)	11 (44%)
Previous ICD-10 diagnoses			
F3× Mood (affective) disorder	23 (46%)	13 (52%)	10 (40%)
F4× Neurotic, stress-related and somatoform disorders	23 (46%)	10 (40%)	13 (52%)
F5× Behavioral syndromes associated with physiological disturbances and physical factors	7 (14%)	3 (12%)	4 (16%)
F6× Disorders of personality and behavior	21 (42%)	10 (40%)	11 (44%)
F8× Pervasive and specific developmental disorders	0 (0%)	0 (0%)	0 (0%)
F9× Behavioral and emotional disorders	9 (18%)	6 (24%)	3 (12%)

Participant gender was assessed by verbally inquiring about their self-identification for the purposes of sample description only.

### Ostracism paradigm manipulation check

**Fig. 2:**
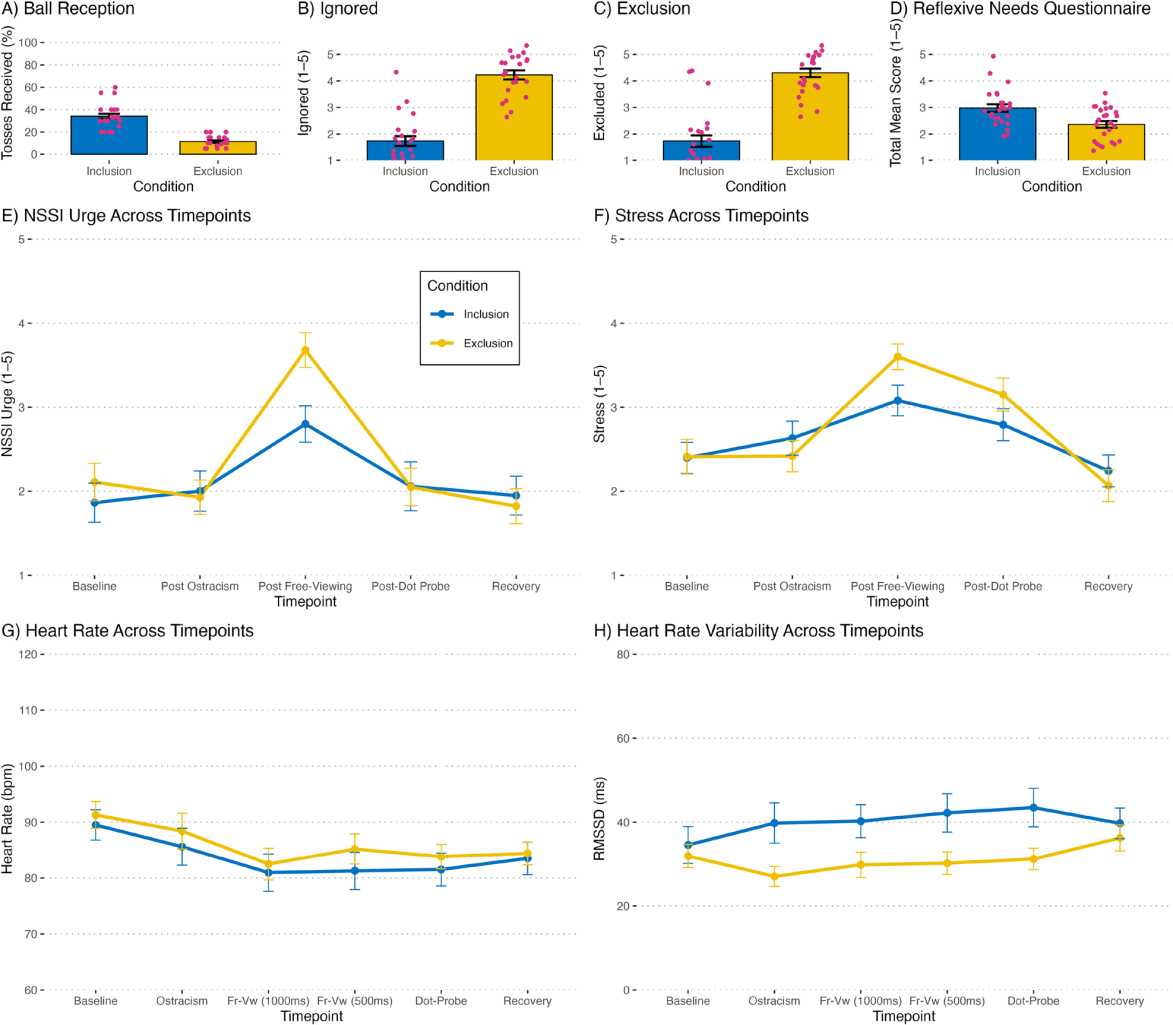
Manipulation check and physiological and subjective trajectories across the experiment. **A–C** In-person ball-tossing ostracism paradigm validation checks, **D** Reflexive Needs Questionnaire, **E–H** Trajectories of urge to engage in NSSI, perceived stress, heart rate, and heart rate variability over the course of the study. Error bars indicate the standard error of the mean (SEM). Fr-Vw Free-Viewing task.

Following the in-person ball-tossing ostracism paradigm, excluded participants reported receiving fewer ball tosses (*t* = 9.53, *p* < 0.001, *g* = 2.74), feeling more ignored (*t* = 9.77, *p* < 0.001, *g* = 2.95) and excluded (*t* = 9.55, *p* < 0.001, *g* = 2.84), and scoring lower on the Reflexive Needs Questionnaire (*t* = 3.28, *p* = 0.002, *g* = 0.91; Fig. 2A–D). These results confirm that the ostracism manipulation successfully induced ostracism at the self-report level. Perceived stress, NSSI urges, and heart rate did not change from baseline, whereas exclusion decreased RMSSD, reflecting reduced vagal activity that remained lower in the exclusion compared to the inclusion condition until the end of the experiment (Fig. 2E–H).

### Attentional bias: free-viewing task

**Fig. 3:**
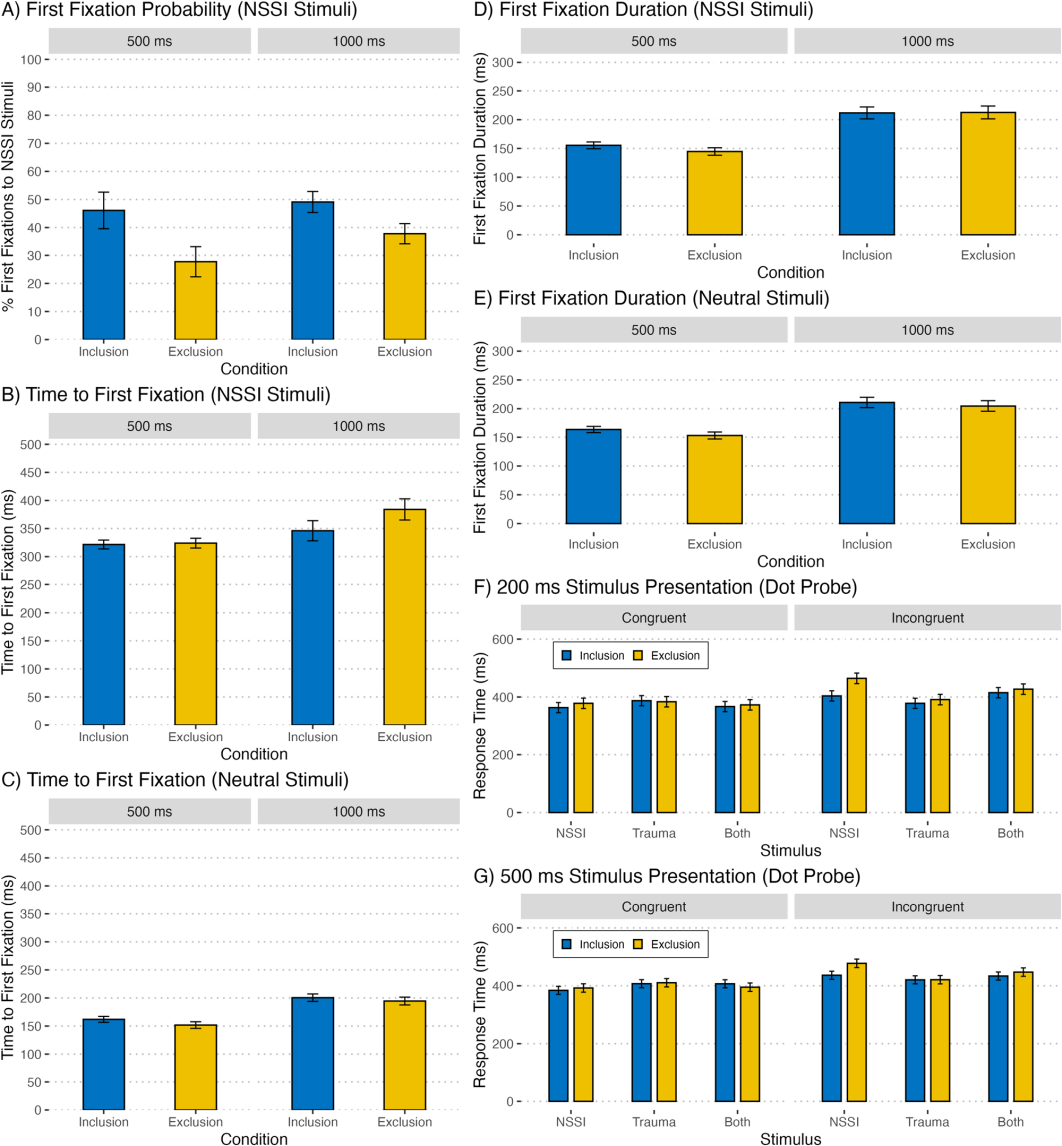
Free-viewing and dot-probe indices of attentional bias. **A–E** Free-viewing task results, **F–G** Response times to congruent and incongruent stimuli in the dot-probe task. Error bars indicate the standard error of the mean (SEM).

During the free-viewing task, heart rate did not differ between conditions, but RMSSD remained lower in the exclusion than the inclusion condition. After the task, NSSI urge and stress increased in both groups, with significantly stronger increases among excluded participants (interactions: heart rate: *F* = 0.39, *p* = 0.585, *R*^2^_m_ = 0.04; RMSSD: *F* = 3.69, *p* = 0.005, *R*^2^_m_ = 0.07; NSSI urge: *F* = 2.46, *p* = 0.047, *R*^2^_m_ = 0.21; stress: *F* = 2.75, *p* = 0.030, *R*^2^_m_ = 0.19). During the free-viewing task, included participants initially fixated on NSSI-related images more often than excluded participants, both at 500 ms (mean difference = 14.88, 95% CI [10.17, 19.68%], *z* = 2.15, *p* = 0.032) and 1000 ms (mean difference = 9.75, 95% CI [4.95, 14.62%], *z* = 2.16, *p* = 0.031), suggesting that ostracism reduced early orienting toward NSSI stimuli. Importantly, both groups exceeded the 25% random chance level of first fixations on NSSI stimuli (as there are always four stimuli shown), indicating attentional bias in both conditions (Fig. 3A).

In contrast, time to first fixation (early orienting latency) did not differ between conditions for NSSI-related (500 ms: *F* = 0.04, *p* = 0.839, *R*²_m_ = 0.00; 1000 ms: *F* = 2.13, *p* = 0.152, *R*²_m_ = 0.02; Fig. 3B) or neutral stimuli (500 ms: *F* = 1.64, *p* = 0.212, *R*²_m_ = 0.00; 1000 ms: *F* = 0.39, *p* = 0.536, *R*²_m_ = 0.00; Fig. 3C), and first fixation duration (sustained engagement) was likewise comparable for NSSI-related (500 ms: *F* = 1.49, *p* = 0.232, *R*²_m_ = 0.01; 1000 ms: *F* = 0.00, *p* = 0.958, *R*²_m_ = 0.00; Fig. 3D) and neutral stimuli (500 ms: *F* = 1.66, *p* = 0.208, *R*²_m_ = 0.00; 1000 ms: *F* = 0.23, *p* = 0.631, *R*²_m_ = 0.00; Fig. 3E), indicating that ostracism affected the probability of initial orienting to NSSI images but not orienting speed (when a fixation occurred) or early dwell time.

### Attentional bias: dot-probe task

No significant differences between conditions were observed in stress, NSSI urge, or heart rate during or following the dot-probe task; only RMSSD remained lower in the exclusion compared to the inclusion condition. Response latencies that deviated more than 3 *SD*s from the mean and incorrect trials were excluded [40], resulting in the removal of 15.58% of trials (6.12% outliers; 9.46% errors; see Supplementary Material for sensitivity analyses without response-time trimming, which yielded a comparable pattern of estimated effects).

Linear mixed-effects models on 200-ms response latencies revealed a significant condition x stimulus x probe interaction (*F* = 5.12, *p* = 0.024, *R*^2^_m_ = 0.04; Fig. 3F). Across conditions, participants responded faster to congruent than to incongruent trials. The interaction was driven by NSSI pairs: excluded participants showed a larger slowing on incongruent relative to congruent trials (86.23 ms, *SE* = 9.63, *p* < 0.001) than included participants (40.43 ms, *SE* = 9.15, *p* = 0.006). No such pattern emerged for trauma pairs (excluded: 7.49 ms, *SE* = 9.38, *p* = 0.999; included: −9.05 ms, *SE* = 9.15, *p* = 0.998). When NSSI and trauma images were presented together, slowing from congruent to incongruent trials was again greater under exclusion than inclusion (54.22 ms, *SE* = 9.43 vs. 47.90 ms, *SE* = 9.21; both *ps* < 0.001).

At 500 ms, the same overall pattern held, with a significant condition x stimulus x probe interaction (*F* = 3.64, *p* = 0.026, *R*^2^_m_ = 0.04; Fig. 3G). Responses were faster on congruent than incongruent trials, and the largest congruent–incongruent difference again appeared for NSSI pairs (excluded: 85.15 ms, *SE* = 10.40, *p* < 0.001; included: 52.28 ms, *SE* = 9.66, *p* < 0.001). Trauma pairs showed no reliable slowing (excluded: 10.49 ms, *SE* = 10.10, *p* = 0.997; included: 13.33 ms, *SE* = 9.59, *p* = 0.976). For concurrent NSSI/trauma, slowing from congruent to incongruent trials was significant under exclusion (52.04 ms, *SE* = 10.40, *p* < 0.001) but not under inclusion (26.85 ms, *SE* = 9.69, *p* = 0.194). Thus, the 500 ms results replicate the 200 ms pattern in a slightly weaker form.

## Discussion

Prior research suggests that engaging in NSSI is associated with attentional bias across adolescence and young adulthood [21, 23]. In this randomized experiment using a live, confederate-based exclusion manipulation (i.e., an adaptation of Cyberball), we found that social exclusion altered autonomic regulation in adolescents with recent NSSI. Exclusion produced sustained vagal withdrawal (lower RMSSD) and was followed by stronger rises in momentary stress and NSSI urges after viewing NSSI images, not immediately after social rejection. The effects of attentional biases were component-specific: although all participants showed an overall bias toward NSSI images (replicating [21]), exclusion attenuated early orienting to NSSI images, whereas in the dot-probe task it selectively magnified incongruency costs, consistent with greater difficulty disengaging (i.e., possibly impaired disengagement). Taken together, acute interpersonal rejection did not simply heighten attention to NSSI cues; instead, our data suggest that ostracism may reorganize attention by dampening initial gaze allocation while degrading attentional efficiency once NSSI information was present, against a backdrop of reduced vagal regulation.

Prior work indicates that social exclusion disproportionately affects adolescents with mental health problems, including those with recent NSSI, chiefly by intensifying negative affect (for a review, see [41]). Neuroimaging research shows that adolescents with NSSI exhibit enhanced ventral anterior cingulate cortex activation following social exclusion in fMRI Cyberball tasks [22], whereas increased dorsomedial prefrontal activation during inclusion was found only in adolescents with Borderline Personality Disorder—compared to those with NSSI or healthy controls [42]. In line with this distinct neural pattern and prior Cyberball studies reporting enhanced negative affect after exclusion in NSSI [43], our manipulation checks confirmed heightened feelings of ostracism and increased negative affect (i.e., thwarted reflexive needs) following exclusion. Contrary to Hypothesis 1, however, exclusion did not produce immediate increases in self-reported stress or NSSI urges, despite the live-interaction Cyberball adaptation intended to improve realism and ecological validity (compare [22, 26, 43, 44]). Physiologically, exclusion produced a clear autonomic shift: RMSSD decreased during exclusion (and remained low), whereas heart rate did not change significantly—consistent with a seated, non-exertional Cyberball context of social-evaluative stress, where mean heart rate may be insufficiently sensitive to subtle autonomic shifts. Taken together, these findings suggest that in adolescents with recent NSSI, ostracism elicits a sustained reduction in parasympathetic/vagal activity (lower RMSSD). This shift is not immediately reflected in self-reports but becomes evident later during subsequent exposure to NSSI imagery, much like everyday encounters on social media [17, 18]. Especially given that the increase in NSSI urge and stress emerged only during later exposure to NSSI imagery, our findings highlight that inter- and intrapersonal processes in NSSI can co-occur and may interact, consistent with functional models of NSSI [19, 45].

With respect to attention, we predicted that exclusion would heighten attentional capture by NSSI images during free-viewing (Hypothesis 2). Instead, exclusion attenuated early orienting to NSSI cues, reflected in a lower first-fixation probability, whereas time to first fixation and first-fixation duration did not differ between conditions. In contrast, Hypothesis 3 was supported: in the dot-probe task, exclusion amplified incongruency costs for NSSI, consistent with greater difficulty disengaging once attention was engaged. Together, these findings indicate stage-dependent effects of exclusion on attentional dynamics, with reduced initial overt selection of disorder-relevant cues but increased later disengagement costs. Within vigilance-avoidance frameworks [30], this pattern suggests that acute interpersonal stress may shift the timing and expression of attentional bias rather than uniformly increasing it. Notably, both conditions still showed an overall bias toward NSSI cues, with first-fixation probabilities exceeding chance levels, consistent with prior eye-tracking work in adolescents with NSSI [21], while exclusion selectively dampened the early gaze-capture component. One conservative account is that exclusion shifts early attentional selection away from NSSI cues because attention is prioritized toward socially salient threat or re-affiliation information [46] and/or because of short-term regulatory avoidance of potentially triggering content. At the same time, sustained parasympathetic withdrawal (lower RMSSD) may bias later-stage processing toward reduced attentional control, resulting in greater difficulty disengaging from high-salience NSSI stimuli. Because our stimulus set did not include social-threat or re-affiliation cues (but see [47], for an example), this mechanism cannot be directly tested here.

Finally, Hypothesis 4 anticipated that exclusion would potentiate subjective stress and NSSI urges and that exposure to NSSI images would further elevate heart rate and decrease RMSSD. As expected, NSSI images increased urges, in both conditions but more so after exclusion. Crucially, after exposure to exclusion, these increases in NSSI urges occurred against the backdrop of already reduced RMSSD rather than being accompanied by additional autonomic change specific to picture exposure, and heart rate remained, again, unchanged. Thus, urge reactivity to NSSI images appears to have been facilitated by the exclusion-induced autonomic context rather than driven by a fresh autonomic response to the images themselves. This aligns with evidence of autonomic dysregulation in NSSI: specifically, reduced vagal tone [29]. Because heart rate variability indexes vagal influence [48] and, via the central autonomic network (prefrontal–limbic circuits), is linked to emotion regulation difficulties [49, 50], it has been of interest in NSSI [51, 52]. Our study adds a temporal account in which exclusion first lowers RMSSD and this parasympathetic state then alters attention (less early capture, greater disengagement difficulty, see [49, 53]) and magnifies NSSI urges during later cue exposure.

Despite several strengths (well-controlled experimental design, carefully recruited clinical sample, and integration of eye-tracking with psychophysiological and self-report measures), several limitations should be noted. Methodological limitations are particularly relevant. First, our live Cyberball adaptation—while effective in eliciting ostracism and negative affect—has not been formally validated against the canonical computerized version [31], which may limit comparability across studies. The NSSI stimuli, previously used [21] and paired with neutral IAPS [34] images, were not drawn from a standardized, normed database and therefore were not validated at scale; validation efforts in this area are only emerging [54]. Although the live Cyberball adaptation and the NSSI stimulus set were intended to increase ecological validity by more closely approximating real-world social exclusion and NSSI-related media exposure, this does not substitute for formal validation of either the task adaptation or the stimulus set. In addition, although RMSSD is a widely used proxy of vagal activity, it is sensitive to factors not fully assessed here, including respiration and ECG artifact burden (e.g., movement-related signal noise, imperfect R-peak detection, ectopic beats), warranting caution in physiological inference [55]. Comorbidities and ongoing treatment may have influenced autonomic and attentional measures, as all participants had at least one outpatient treatment contact and 46% were taking psychotropic medication at the time of testing, while the sample size precluded adequately powered covariate-adjusted analyses. Medication heterogeneity may have contributed additional variability to both physiological and attentional outcomes. Conceptual and interpretive limitations should also be considered. Importantly, without a non-NSSI/healthy control group, we cannot determine whether the observed autonomic and attentional patterns are specific to adolescents with recent NSSI or instead reflect more general effects of social exclusion. Finally, because the sample was predominantly female and gender-diverse (a distribution typical of this population [56, 57]) and largely treatment-engaged, generalizability to males, non-treatment-seeking youth, young adults, and community samples may be limited. Because the study may have been underpowered for smaller effects, larger, well-powered studies—including appropriate control groups and ecologically relevant, formally validated designs—are needed to test the robustness and specificity of these effects.

Taken together, our findings have implications for prevention and intervention in online and clinical contexts. In online environments, they underscore the potential value of content warnings and active moderation to limit unsolicited exposure to NSSI-related imagery [12]. Schools and caregivers may complement this with social-media literacy guidance and programs that foster social inclusion and anti-bullying resilience. Clinically, our study supports routine assessment of recent ostracism (online and offline) and exposure to NSSI-related imagery on social media, as well as post-stressor monitoring and safety planning. Skills that target autonomic regulation, such as paced breathing, relaxation training, and heart-rate variability biofeedback, may be reasonable adjuncts [58, 59]. Building on this, future research should test interventions that more directly address autonomic imbalance, for example transcutaneous auricular vagus nerve stimulation (taVNS), which can enhance parasympathetic tone and modulate central autonomic networks [60] if our findings are replicated. Early evidence in PTSD and depression supports feasibility [61, 62], warranting efficacy trials in self-injuring populations given heart-rate variability’s potential as an actionable target to reduce NSSI urges, and overall clinical burden.

## Conclusion

In conclusion, we show that among adolescents who engage in NSSI, social rejection is followed by sustained parasympathetic withdrawal that later coincides with later-stage (disengagement-related) attentional difficulty—rather than increased initial capture—toward NSSI-related images, and with heightened urges to self-injure.

## Supplementary information

Supplementary Material

## Data Availability

De-identified participant data for research purposes are available on request only due to restrictions laid out in the study’s participant consent forms and the Medical University of Vienna’s data-protection policies.

## Code availability

Code used for analyses is available on request from the corresponding author, without restriction.
