# Supplementary material for: Social exclusion alters attention and autonomic regulation in adolescents with nonsuicidal self-injury
Source: Transl Psychiatry. 2026 May 24;16:367. doi: 10.1038/s41398-026-04136-w (PMC13376568; doi:10.1038/s41398-026-04136-w)
Supplement: Supplementary file 1 — Supplementary Material [file 41398_2026_4136_MOESM1_ESM.docx]

**Supplementary Material**

**Model Diagnostics for Mixed-Effects Models**

All inferential analyses used mixed-effects models with random intercepts for participants (ID) estimated in lme4 (LMMs via lmer, GLMMs via glmer; binomial-logit for fixation probability). To increase transparency, we conducted a standardized diagnostic workflow for all mixed models reported in the manuscript, including (i) self-report/physiology outcomes (NSSI urge, stress, heart rate, RMSSD), (ii) eye-tracking outcomes (first-fixation probability, time to first fixation [TTFF], first-fixation duration; each at 500 ms and 1000 ms exposure), and (iii) dot-probe response-time outcomes (200 ms and 500 ms). For every model, we checked estimation integrity (singularity, optimizer convergence status, and maximum absolute gradient from the optimizer) and inspected model assumptions graphically. For LMMs, we generated residual Q–Q plots and residuals vs. fitted plots. For binomial GLMMs, we used simulation-based residual diagnostics (DHARMa), including uniformity Q–Q plots and scaled residuals vs. predicted values, complemented by DHARMa tests for uniformity, dispersion, and outliers.

Across the full set of multilevel models, no singular fits were detected and models converged without warnings, indicating stable estimation for the specified random-intercept structure. Optimizer gradients were small for all LMMs (maximum absolute gradient ≤ 3.75 × 10⁻⁵ across the eye-tracking/dot-probe LMMs; and ≤ 1.26 × 10⁻⁶ for the self-report/physiology LMMs). For the binomial GLMMs of first-fixation probability, maximum absolute gradients were < 1 × 10⁻³, consistent with acceptable convergence. Visual diagnostics (Supplementary Figures 1–2) indicated that LMM residuals were generally well-behaved; mild tail deviations in Q–Q plots were most evident for reaction-time and fixation-duration measures (expected for positively skewed latency distributions and residual extremes even after trimming), while residuals-versus-fitted plots did not suggest pronounced nonlinearity or strong heteroscedasticity patterns. For the fixation-probability GLMMs, DHARMa diagnostics did not indicate meaningful misspecification (uniformity p = .053 [500 ms] and .566 [1000 ms]; dispersion p = .344 and .708; outlier p = 1.00 and .06), supporting the adequacy of the binomial-logit specification for inference.

**Supplementary Figure 1.** LMM diagnostics for self-report and physiological outcomes. Residual Q–Q plots and residuals versus fitted values for the LMMs of NSSI urge, stress, heart rate, and RMSSD (each with random intercepts by participant).


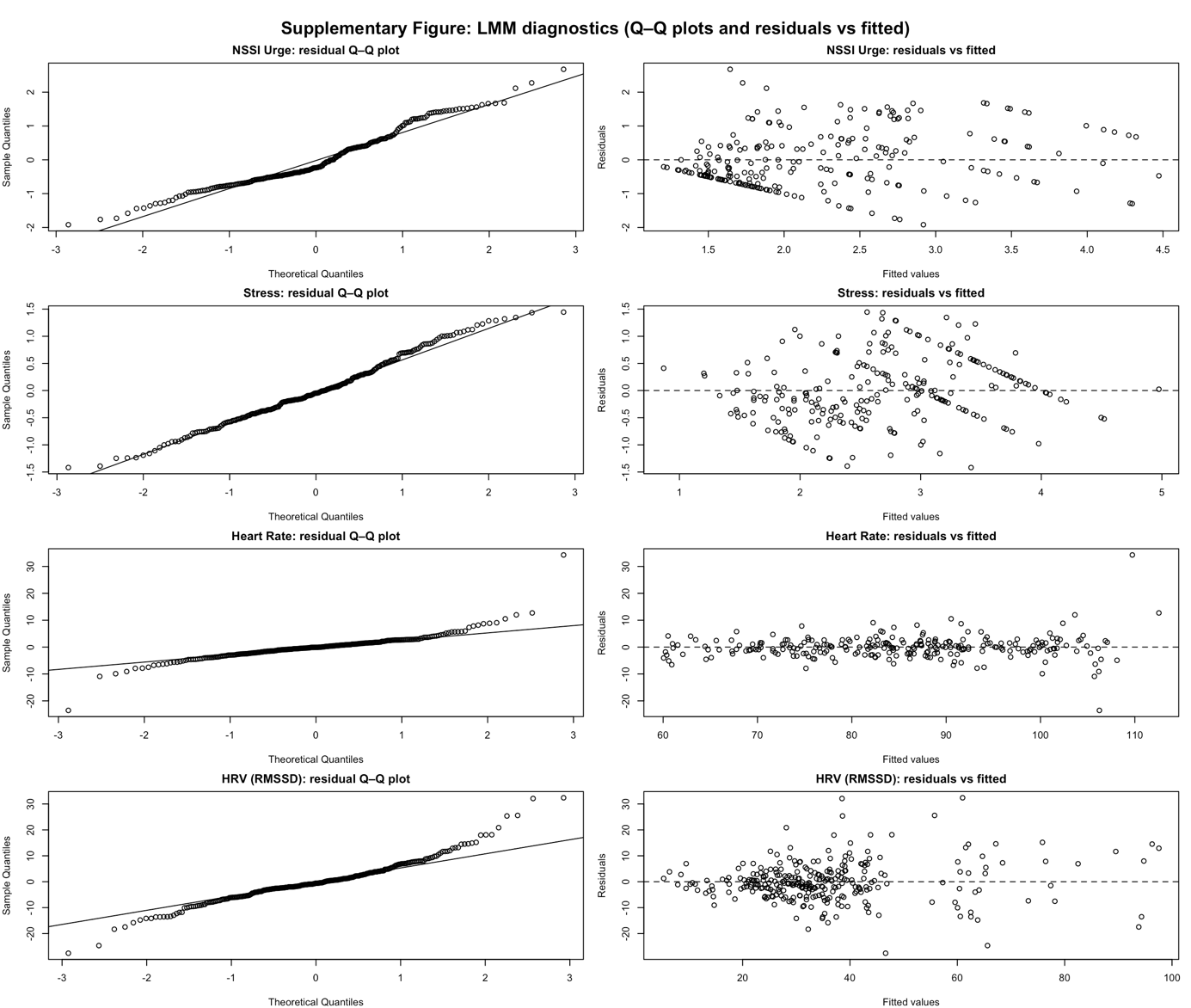


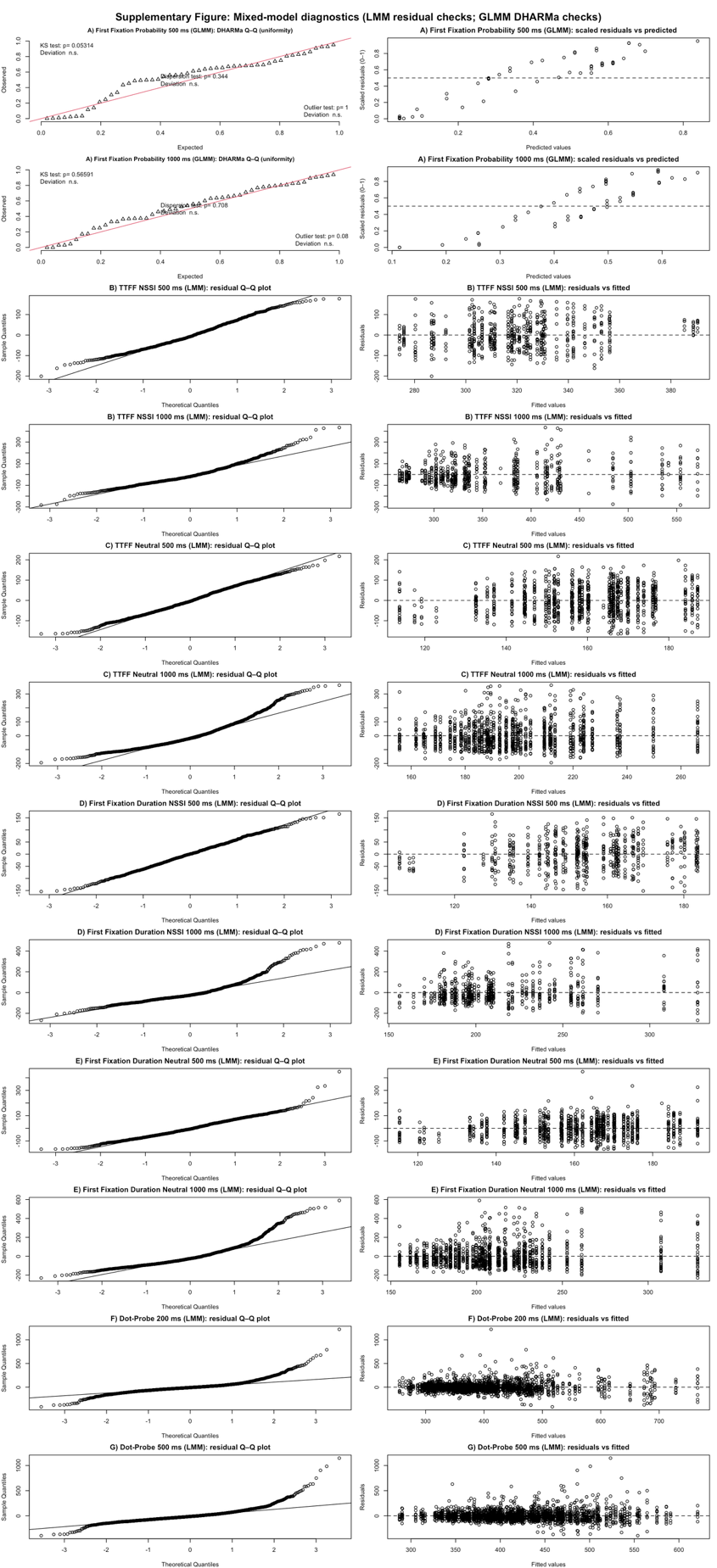
**Supplementary Figure 2.** Mixed-model diagnostics for eye-tracking and dot-probe outcomes. For binomial GLMMs of first-fixation probability (500 ms, 1000 ms), DHARMa uniformity Q–Q plots and scaled residuals versus predicted values are shown. For LMMs of TTFF (NSSI, neutral; 500/1000 ms), first-fixation duration (NSSI, neutral; 500/1000 ms), and dot-probe RTs (200 ms, 500 ms), residual Q–Q plots and residuals versus fitted values are shown.

**Sensitivity Analysis: Dot-Probe Reaction Time Outliers**

In the main analyses of our manuscript, we applied an outlier-screening procedure to reduce the influence of extreme reaction times that likely reflect attentional lapses, transient disengagement from the task, or other non-task-related factors. Specifically, within each participant and condition cell (defined by probe congruency and stimulus category), reaction times deviating more than ±3 *SD* from the cell mean were excluded prior to mixed-effects modeling (consistent with guidelines regarding the dot-probe paradigm^1^). The sensitivity analysis was based on 2,875 (200 ms) and 2,775 (500 ms) individual data points, whereas the original analysis was based on 2,709 (200 ms) and 2,615 (500 ms) data points.

To evaluate the robustness of findings to outlier handling, we conducted a sensitivity analysis in which the same models were re-estimated without excluding reaction-time outliers (i.e., retaining all correct trials). In this untrimmed dataset, the Condition × Congruency × Stimulus interaction remained significant at both presentation durations (200 ms: *F* = 5.96, *p* = .003, *R*^2^_m_ = .04; 500 ms: *F* = 3.51, *p* = .030, *R*^2^_m_ = .03), despite increased residual variance when extreme response times are retained. The pattern of estimated incongruency costs remained comparable to the primary analysis. At 200 ms, excluded participants showed a larger slowing on incongruent relative to congruent trials for NSSI pairs (104.09 ms, *SE* = 11.80, *p* < .001) than included participants (31.80 ms, *SE* = 11.30, *p* = .172), whereas trauma pairs showed no reliable slowing (excluded: 25.16 ms, *SE* = 11.50, *p* = .560; included: −10.70 ms, *SE* = 11.00, *p* = .998). When NSSI and trauma images were presented together, incongruency costs were again significant in both conditions (excluded: 51.46 ms, *SE* = 11.50, *p* = .001; included: 58.20 ms, *SE* = 11.30, *p* < .001).

At 500 ms, the same descriptive pattern was observed, with larger incongruency costs for NSSI pairs under exclusion (96.65 ms, *SE* = 13.50, *p* < .001) than inclusion (51.85 ms, *SE* = 12.50, *p* = .002), no reliable slowing for trauma pairs (excluded: 4.94 ms, *SE* = 13.10, *p* = .999; included: 21.82 ms, *SE* = 12.60, *p* = .853), and significant slowing for NSSI/trauma pairs under exclusion (52.16 ms, *SE* = 13.60, *p* < .001) but not inclusion (12.41 ms, *SE* = 12.70, *p* = .1998).

Overall, retaining all correct trials (i.e., no response time outlier trimming) preserved the direction and (most often) magnitude of the estimated incongruency-cost pattern found in the main analyses (larger costs for NSSI under exclusion) but increased residual variance and shifted point estimates.

**References**

1 Price RB, Kuckertz JM, Siegle GJ, Ladouceur CD, Silk JS, Ryan ND *et al.* Empirical recommendations for improving the stability of the dot-probe task in clinical research. *Psychological Assessment* 2015; **27**: 365–376.
